# Patterns and Prevalence of Food Allergen Cross-Reactivity: A Cross-Sectional Study of Food-Allergic Adults in the Makkah Region, Saudi Arabia

**DOI:** 10.7759/cureus.82942

**Published:** 2025-04-24

**Authors:** Amna Alotiby

**Affiliations:** 1 Department of Haematology and Immunology, College of Medicine, Umm Al-Qura University, Makkah, SAU

**Keywords:** allergen sensitization, atypical allergen patterns, co-sensitization, cross-reactivity, food allergy, saudi arabia

## Abstract

Background and objectives: Food allergies are a growing public health concern, with cross-reactivity complicating diagnosis and management. Cross-reactivity occurs when immune responses to one allergen trigger reactions to similar proteins in other allergens. There is a lack of regional data on the prevalence and patterns of food allergen cross-reactivity in Saudi Arabia, particularly in the Makkah region. This study investigates the prevalence and patterns of food allergen cross-reactivity among adults in Makkah, considering gender- and age-related differences.

Methods: A cross-sectional survey was conducted between July and October 2021 among 93 adults aged 18 years or older residing in the Makkah region who self-reported doctor-diagnosed food allergies. Participants were recruited through an online questionnaire distributed via social media. The survey, validated by expert reviewers and pretested for clarity, collected demographic information, allergen types, and cross-reactivity data. Descriptive statistics, chi-square tests, and a cross-reactivity matrix were used to assess allergen co-sensitization patterns and demographic associations.

Results: The study identified high rates of nut, egg, and fruit allergies, with significant cross-reactivity, particularly among participants allergic to nuts. Females reported higher allergy prevalence of cross-reactivity (65.6%) compared to males (34.4%), aligning with global trends. Middle-aged females exhibited the highest rates of allergen cross-reactivity. The most frequent cross-reactivity was observed in participants with nut (100%), egg (90.7%), and fruit (89.2%) allergies. Specifically, 90.9% of individuals allergic to nuts experienced cross-reactivity with more than two other allergens. The cross-reactivity matrix revealed both expected (e.g., between tree nuts and peanuts) and unexpected patterns (e.g., between fruits and seafood), suggesting the possible influence of panallergens, co-sensitization, or regional dietary factors.

Conclusions: This study highlights a high prevalence of food allergen cross-reactivity among adults in the Makkah region, with significant gender- and age-related trends. The findings underscore the need for clinician awareness of atypical and panallergen-driven cross-reactivity to support tailored patient counseling and management strategies. Region-specific screening protocols and culturally informed public health campaigns are recommended to address local dietary practices and improve allergy awareness. Future longitudinal and interventional studies incorporating objective diagnostic tools are essential to validate these findings and assess the effectiveness of targeted interventions. These insights can inform clinical guidelines and national health policies to improve diagnosis, treatment, and patient education in Saudi Arabia.

## Introduction

Food allergies represent a significant and growing public health concern worldwide, with prevalence rates steadily increasing in recent decades across children and adults in both developed and developing countries [1]. These allergies can provoke various immune responses, from mild skin rashes and gastrointestinal issues to severe and life-threatening reactions such as anaphylaxis. This growing burden not only affects the quality of life of individuals but also places considerable strain on healthcare systems, necessitating ongoing management, preventive strategies, and emergency interventions [2]. In Saudi Arabia, the burden of food allergies is increasingly recognized. Recent national estimates report adult food allergy prevalence between 17% and 22% [3]. More specifically, a cross-sectional study conducted in the Makkah region found that 17.5% of Saudi adults reported food allergies, with seafood, eggs, and milk identified as the most common allergens [4].

A notable challenge in managing food allergies is cross-reactivity - a phenomenon where an individual's immune system responds to allergens across multiple, structurally similar foods. This introduces complexities that can complicate both diagnosis and treatment [5,6]. Cross-reactivity is primarily mediated by IgE antibodies and occurs when allergens share homologous protein structures. Known cross-reactive protein families include profilins, pathogenesis-related (PR-10) proteins, lipid transfer proteins (LTPs), and Bet v1 homologs, which are frequently implicated in reactions involving plant-based foods and pollens [7].

While this phenomenon is well-documented between certain types of allergens, such as those found in tree nuts and legumes, the comprehensive evaluation of cross-reactivity among a broader range of food allergens in adults remains underexplored [5-7]. Previous research has generally focused on a limited set of allergens, with insufficient attention to the broader patterns and frequency of cross-reactivity that might inform more accurate diagnostics and effective dietary management [2,8-10]. Most of these studies have examined food allergies and cross-reactivity patterns in Western populations; however, regional variations in allergen types and cross-reactivity have not been fully explored in non-Western contexts, including the Middle East. Specifically, in the Makkah region of Saudi Arabia, food consumption patterns, such as a high intake of seafood, dates, and traditional grains, and environmental exposures, such as dust storms and regional pollens, may influence the prevalence and types of food allergens, as well as cross-reactivity patterns [11-13].

Understanding these specificities is crucial for developing targeted interventions, public health strategies, and improved clinical guidelines tailored to the population. To our knowledge, this is the first study in Saudi Arabia to analyze allergen cross-reactivity patterns using a matrix-based approach, specifically within the adult population of the Makkah region. This approach uses a cross-tabulated matrix to identify how often different food allergens co-occur, helping reveal cross-reactive clusters and sensitization patterns within the population. Therefore, this study aims to fill this knowledge gap by assessing the prevalence and patterns of food allergen cross-reactivity among adult residents of the Makkah region. By exploring demographic trends and examining associations among commonly encountered food allergens, the study provides insights into the unique allergen landscape of the region, contributing valuable data to inform better allergy management practices for the Saudi population.

## Materials and methods

Study design and population

This study employs a cross-sectional survey research design conducted within the geographical confines of the Makkah region in Saudi Arabia. The data were collected online using a Google Forms questionnaire distributed via social media platforms from 08 July 2021 to 01 October 2021. The focus of this research is on individuals who suffer from food allergies. The participants included in the study were adults aged 18 years or older who self-reported having a history of food allergies and confirmed that a doctor had diagnosed their food allergies. The study aimed to explore the prevalence and patterns of food-allergen cross-reactivity among food-allergic adults, considering gender- and age-related differences. This study serves as an initial screening tool to assess the prevalence and patterns of food-allergen cross-reactivity in food-allergic adults. Subsequently, a survey-based approach was preferred because it enabled the collection of broad, community-level data and is expected to inform the design of future studies involving clinical testing.

In the process of determining the suitable sample size for this study, a thorough review of two prior investigations conducted in the Makkah region was undertaken. These earlier studies reported a range of 70-100 individuals diagnosed with food allergies within the specified geographical area [3,14]. Drawing from this information available in the existing literature, the required sample size for the present study was estimated using OpenEpi software (version 3.0; Centers for Disease Control and Prevention, Atlanta, GA) [15]. Based on the calculated parameters, a minimum sample size of 80 was estimated to provide adequate representativeness and exploratory power for descriptive analysis. This estimation assumed a 5% margin of error and a 95% confidence level for a small population. However, during the actual implementation of the research, the study ultimately enrolled a total of 93 participants.

The exclusion criteria included individuals under 18 years of age and those without a history of food allergies or who did not confirm that a doctor had diagnosed their food allergies. The study received ethical approval from the Biomedical Ethics Committee, Umm Al-Qura University, with approval number HAPO-02-K-012-2021-03-631, dated 18 March 2021.

Data collection

For data collection, a structured questionnaire was developed, inspired by previous studies, and comprising three sections [3,4,11]. The first section acts as a screening tool, where participants are asked whether they have a history of food allergies and whether a doctor has formally diagnosed them. Participants who affirm both conditions proceed to the next section, which outlines the inclusion criteria. The second section gathers demographic information, such as age and gender, from participants who meet the inclusion criteria. The third section is designed to assess both the presence of food allergies and the potential for cross-reactivity. In this section, participants are asked to report all food allergens to which they are allergic.

The questionnaire was pretested with a small sample of participants to ensure the clarity and relevance of the questions. Its validity was confirmed through expert review, ensuring that the questions aligned with the study's objectives. Additionally, internal consistency was assessed using Cronbach's alpha, which resulted in a coefficient of 0.87, indicating excellent reliability. This further supports the reliability of the instrument in capturing accurate and consistent data.

Data analysis

Data were entered and analyzed using Statistical Product and Service Solutions (SPSS, version 25; IBM SPSS Statistics for Windows, Armonk, NY). Descriptive analysis based on frequency and percent distribution was performed for all variables, including participants' demographic data and the most reported allergens. Food allergen cross-reactivity was tabulated based on the number of cross-reactive allergens reported by each participant. A cross-reactivity matrix was then constructed electronically using the same software (SPSS) to assess the probability of cross-reactivity among the different reported food allergens. The matrix was created by recording the co-occurrence of allergen pairs that participants reported together. Each cell in the matrix represented the frequency of co-reported allergen pairs, which was then used to calculate the likelihood of cross-reactivity. Chi-square tests were used to evaluate associations between categorical variables, such as demographic characteristics and allergen reporting patterns, as well as to examine statistically significant trends in cross-reactivity. Statistical significance was set at p<0.05.

## Results

Participant demographics

A total of 93 adults with food allergies were enrolled in the study. The sample had a higher proportion of females (65.6%) compared to males (34.4%). The age distribution was as follows: 28% of participants were aged 18-28 years, 18.3% were aged 29-39, 37.6% were aged 40-59, and 16.1% were aged 60-80 (Table 1).

**Table 1:** Demographic characteristics of food-allergic adults in the Makkah Region, Saudi Arabia.

Personal Data	No.	%
Age (years)		
18-28	26	28.0%
29-39	17	18.3%
40-59	35	37.6%
60-80	15	16.1%
Gender		
Male	32	34.4%
Female	61	65.6%

Food allergen cross-reactivity prevalence and frequency among the participants

**Figure 1:**
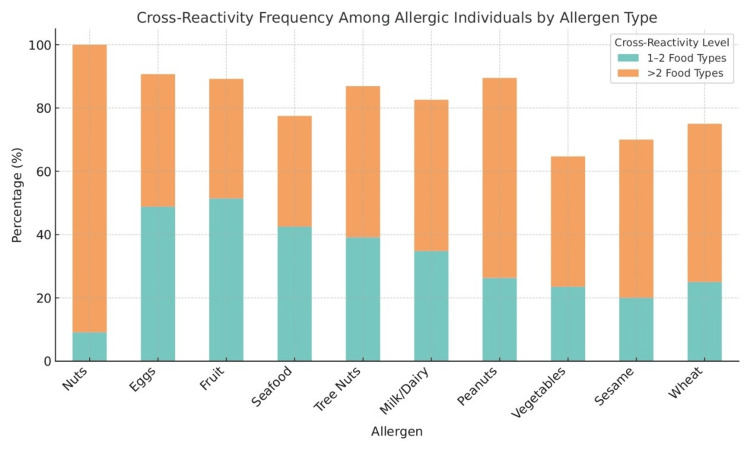
Stacked bar chart illustrating the distribution of food allergen cross-reactivity among participants. For each allergen category, the chart shows the percentage of individuals who reported being allergic to one to two food types (limited cross-reactivity) versus those allergic to more than two food types (extensive cross-reactivity), based on the subgroup of individuals allergic to each specific food.

The prevalence of specific food allergens and their frequencies of cross-reactivity among the participants are illustrated in Table 2 and visualized in Figure 1. Overall, a considerable percentage of participants experienced cross-reactivity with multiple allergens, particularly those allergic to nuts, eggs, and fruits. This analysis distinguishes between participants who reported being allergic to one to two food types (limited cross-reactivity) and those with more than two food types (extensive cross-reactivity).

**Table 2:** Cross-reactivity frequency among allergic individuals by allergen type.

Allergen	1–2 Food Types Cross-Reactivity (%)	>2 Food Types Cross-Reactivity (%)	Overall, Cross-Reactivity (%)
Nuts	9.1%	90.9%	100.0%
Eggs	48.8%	41.9%	90.7%
Fruit	51.4%	37.8%	89.2%
Seafood	42.5%	35.0%	77.5%
Tree Nuts	39.1%	47.8%	87.0%
Milk/Dairy	34.8%	47.8%	82.6%
Peanuts	26.3%	63.2%	89.5%
Vegetables	23.5%	41.2%	64.7%
Sesame	20.0%	50.0%	70.0%
Wheat	25.0%	50.0%	75.0%

Nuts emerged as the allergen with the highest likelihood of extensive cross-reactivity, with 90.9% of affected individuals reporting reactions to more than two other food types, indicating complex allergic profiles. Peanuts (63.2%) and milk/dairy (47.8%) also showed high rates of broader cross-reactivity. In contrast, allergens such as eggs (48.8%), fruit (51.4%), and seafood (42.5%) were associated with more limited cross-reactivity, though still substantial in prevalence.

The overall prevalence of cross-reactivity was highest for nuts (100.0%), followed by eggs (90.7%), peanuts (89.5%), and fruit (89.2%). Lower rates were observed among individuals allergic to vegetables (64.7%), sesame (70.0%), and wheat (75.0%). These findings indicate that individuals with these specific food allergies may be at heightened risk for co-sensitization and should be monitored closely for dietary cross-reactions.

The stacked bar chart in Figure 1 visually highlights the distribution of limited and extensive cross-reactivity across allergen types. These patterns underscore the complexity of managing food allergies in this population, as individuals often exhibit broad sensitivities that may require personalized dietary guidance and avoidance strategies.

Chi-square tests were conducted to assess statistical differences in cross-reactivity frequencies across allergen groups. Percentages were calculated based on the total number of individuals reporting each allergen, using subgroup-based calculations to represent cross-reactivity within each allergen group accurately.

Association between demographic factors and cross-reactivity frequency

**Figure 2:**
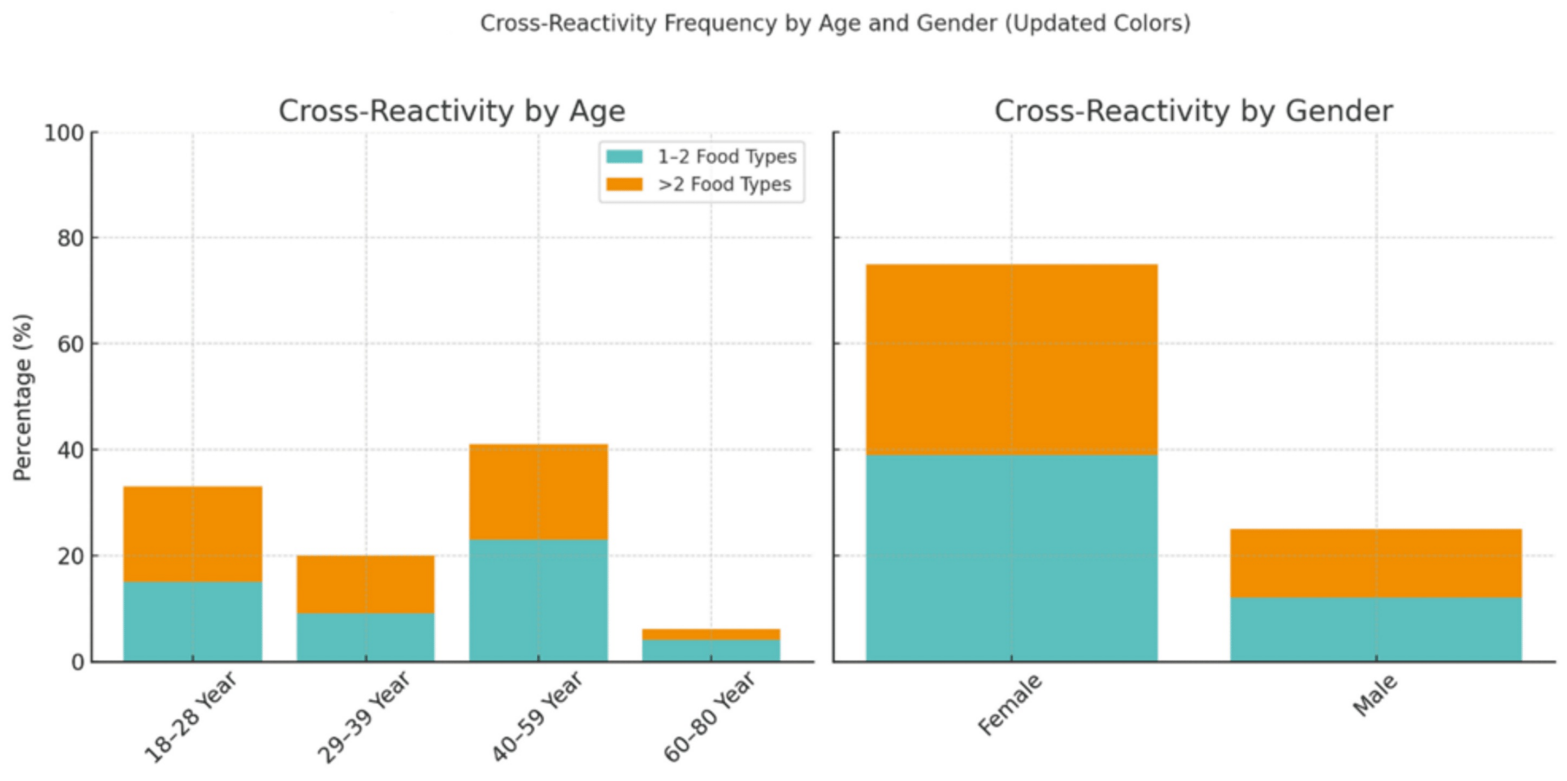
Stacked bar charts illustrating the frequency of cross-reactivity among allergic individuals by demographic factors (age and gender). The charts display the proportion of individuals experiencing limited (one to two food types) and extensive (>2 food types) cross-reactivity within each group.

The association between demographic characteristics and the frequency of food allergen cross-reactivity is presented in Table 3 and visualized in Figure 2. Cross-reactivity levels were categorized into limited (one to two food types) and extensive (>2 food types) to explore potential patterns across different age groups and between genders.

**Table 3:** Association between demographic factors and cross-reactivity frequency.

Variable	Category	1–2 Food Types	>2 Food Types	p-value
Age	18–28 Year	15%	18%	p = 0.093
	29–39 Year	9%	11%	
	40–59 Year	23%	18%	
	60–80 Year	4%	2%	
Gender	Female	39%	36%	p = 0.029
	Male	12%	13%	

Gender was significantly associated with the extent of food allergen cross-reactivity (p = 0.029). As illustrated in Figure 2, females accounted for the majority of participants reporting food allergy cross-reactivity. Among females, both limited (39%) and extensive (36%) cross-reactivity were notably more prevalent compared to males (12% and 13%, respectively). This trend suggests that females may be more susceptible to broader sensitization and experience more complex allergic profiles.

In contrast, age was not significantly associated with cross-reactivity frequency (p = 0.093), though certain patterns were observed. Middle-aged individuals (40-59 years) showed the highest overall frequency of cross-reactivity (23% limited, 18% extensive), followed by the youngest group (18-28 years), who exhibited a slightly higher percentage of extensive (18%) than limited (15%) cross-reactivity. Older age groups (60-80 years) had the lowest levels of cross-reactivity, potentially reflecting reduced immune sensitization or dietary diversity with age.

These observations indicate that females and middle-aged adults are more likely to report food allergen cross-reactivity, with a considerable proportion of them experiencing extensive reactions. Understanding these demographic patterns is essential for improving individualized allergy assessments and dietary management strategies.

Food allergen cross-reactivity pattern among the participants

**Figure 3:**
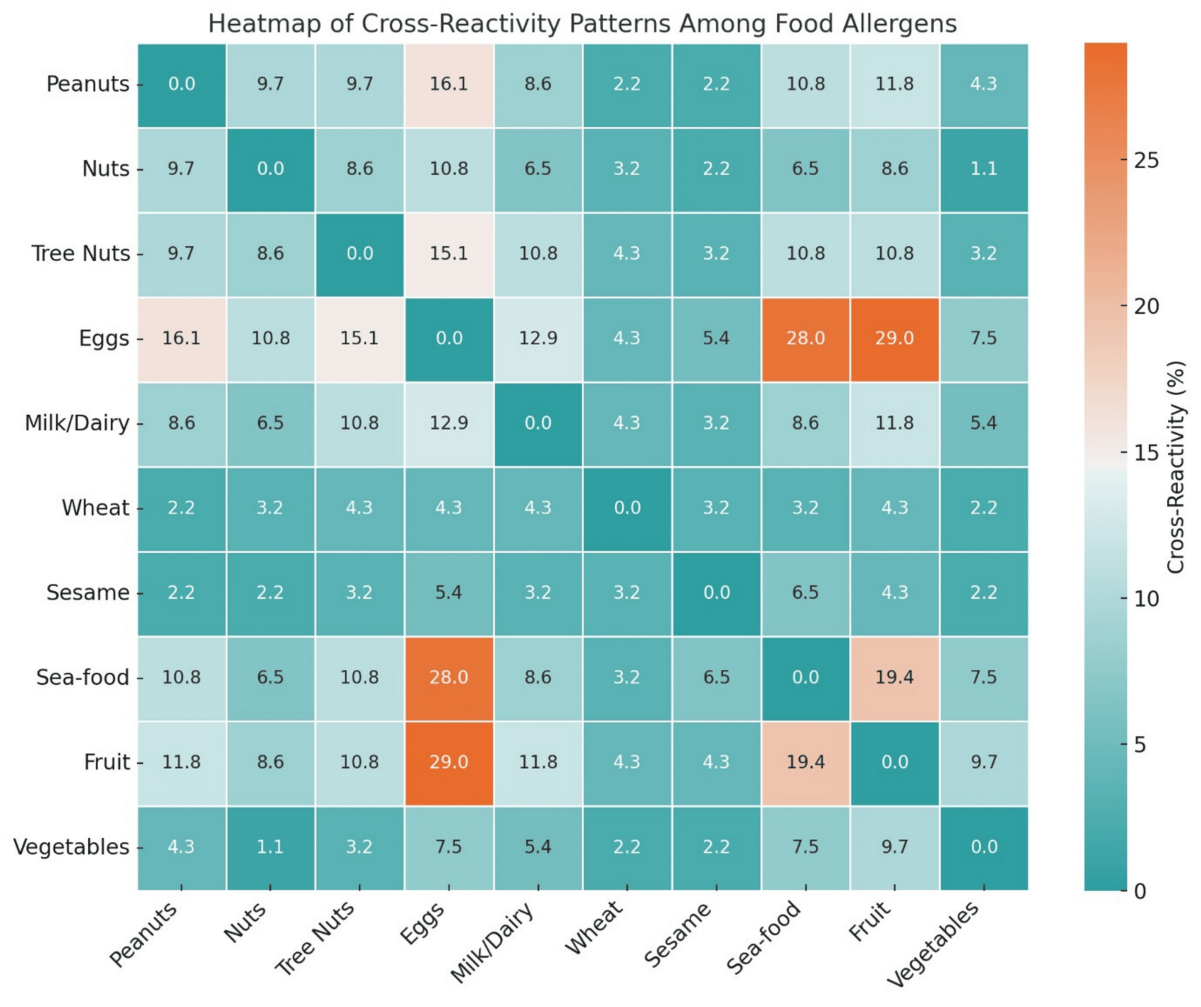
Heatmap of cross-reactivity patterns among food allergens. This figure illustrates the percentage of cross-reactivity between various food allergens as reported by participants. Darker shades indicate stronger cross-reactivity. Notably, eggs, fruit, and seafood exhibit higher cross-reactivity percentages with multiple other allergens, suggesting complex allergenic profiles. The symmetric matrix highlights co-sensitization patterns that are important for clinical evaluation and dietary management.

The patterns of cross-reactivity among different food allergens are presented in Table 4 and visualized in Figure 3. These findings reveal substantial overlap in sensitization across multiple food groups among participants, suggesting the presence of immunological cross-reactivity or co-sensitization.

**Table 4:** Cross-reactivity patterns among food allergens.

Food Allergens	Peanuts	Nuts	Tree Nuts	Eggs	Milk/Dairy	Wheat	Sesame	Seafood	Fruit	Vegetables
Peanuts	-									
Nuts	9.7%									
Tree Nuts	9.7%	8.6%	-							
Eggs	16.1%	10.8%	15.1%	-						
Milk/Dairy	8.6%	6.5%	10.8%	12.9%	-					
Wheat	2.2%	3.2%	4.3%	4.3%	4.3%	-				
Sesame	2.2%	2.2%	3.2%	5.4%	3.2%	3.2%	-			
Seafood	10.8%	6.5%	10.8%	28%	8.6%	3.2%	6.5%	-		
Fruit	11.8%	8.6%	10.8%	29%	11.8%	4.3%	4.3%	19.4%	-	
Vegetables	4.3%	1.1%	3.2%	7.5%	5.4%	2.2%	2.2%	7.5%	9.7%	-

High levels of cross-reactivity were observed between eggs and fruit (29%), eggs and seafood (28%), and fruit and seafood (19.4%), indicating that individuals allergic to eggs or fruits are more likely to react to other allergens as well. Additionally, tree nuts and peanuts (9.7%) and tree nuts and milk/dairy (10.8%) showed notable associations.

Across the heatmap, eggs appeared as a central allergen with moderate-to-high reactivity across most food groups, including milk/dairy (12.9%), tree nuts (15.1%), and nuts (10.8%). Similarly, fruits and seafood showed broader patterns of cross-reactivity with other allergens, such as vegetables and dairy, reflecting complex allergic responses.

While some allergens, such as wheat, sesame, and vegetables, exhibited lower overall cross-reactivity percentages, they still demonstrated consistent connections with other groups, especially in participants with extensive food allergies. These patterns may reflect shared allergenic proteins (e.g., profilins or lipid transfer proteins) or common exposure pathways in the local diet.

Overall, this analysis underscores the clinical importance of assessing cross-reactivity patterns in food-allergic individuals. It highlights the need for comprehensive diagnostic evaluations and careful dietary planning, particularly for individuals reporting multiple food allergies or high-risk allergens such as eggs, fruits, and seafood.

## Discussion

This study aimed to explore the prevalence and cross-reactivity patterns of food allergies among adults in the Makkah region. This analysis provides valuable insights into the prevalence of food allergies and cross-reactivity patterns among adults in the Makkah region, shedding light on the broader tendency for individuals with allergies to one food type to develop sensitivities to other food allergens. Consistent with findings from global research, fruits, eggs, and seafood emerged as the most common allergens, with notable cross-reactivity rates, particularly among those with nut allergies. Specifically, over 90% of participants with nut allergies reported cross-reactivity with other foods, indicating that structural similarities between nut proteins and other allergenic proteins may be prominent in this population, posing challenges for dietary management and patient education [2,16]. This may be attributed to shared allergenic epitopes, such as vicilins, legumins, and 2S albumins, commonly found in nuts and seeds. Such homology can lead to IgE binding across different allergens, contributing to clinically significant cross-reactivity [17].

In the present study, significant cross-reactivity was observed between tree nuts and other common allergens, such as eggs and dairy products. This finding aligns with previous research, which has consistently highlighted the importance of considering multiple allergens in the diagnostic process, particularly when a patient presents with a history of tree nut allergies. Studies by Sicherer et al. [9] and Cox et al. [16] emphasize that individuals sensitized to one allergen may also be at risk for developing sensitivities to related proteins, such as those found in milk or eggs.

Based on these findings, clinicians in regions with similar dietary exposures may benefit from broader allergen testing panels when evaluating individuals with known nut allergies. Incorporating cross-reactivity data into allergy screening protocols can help identify potential co-sensitizations early and guide safer dietary planning. For instance, a history of nut allergy may warrant preemptive screening for sensitivity to legumes, seeds, or dairy when supported by patient symptoms or family history.

Interestingly, some cross-reactivity patterns observed in this study, such as those between peanuts and eggs or between fruits and seafood, may diverge from classical IgE-mediated allergy groupings [16,18]. However, such findings are not unprecedented and may reflect co-sensitization due to shared dietary exposures, atopic predisposition, or the presence of common panallergens such as profilins or tropomyosins. Previous literature has documented similar patterns in polyallergic individuals, supporting the complexity and heterogeneity of food allergy profiles in clinical populations [9,16,19].

Furthermore, the development of a cross-reactivity matrix in this study provides valuable insight into the specific associations between tree nuts and other allergens, which could inform future clinical guidelines for allergy testing and management. The findings also underscore the need for region-specific research, as local dietary habits and environmental factors are likely to influence allergy profiles [12]. In the context of Makkah, traditional diets often include high consumption of dates, tahini (sesame paste), mixed nuts, and seafood-based dishes such as sayadiyah, particularly during cultural and religious gatherings [11]. These dietary staples may contribute to early sensitization and sustained allergen exposure. For example, dates are commonly consumed alongside nuts, a combination that could increase the risk of co-sensitization due to repeated and simultaneous exposure to allergenic proteins.

Previous literature on geographic variations in food allergy prevalence, such as the work by Peters et al. [12] and Bartra et al. [13], supports the notion that allergy profiles can differ considerably by region, further highlighting the importance of conducting localized research better to understand the specific allergenic risks in different populations. Integrating these findings into clinical practice could lead to more accurate and comprehensive allergy diagnoses, ultimately improving patient outcomes by reducing the risk of overlooked co-sensitizations and improving the overall management of food allergies.

These demographic patterns highlight a need for gender- and age-specific screening and education strategies. Notably, females showed a higher prevalence of reported allergies, aligning with global studies that suggest that hormonal factors and health-seeking behaviors contribute to gender differences in allergy rates. Hormonal fluctuations, particularly during puberty, pregnancy, and menopause, have been shown to influence immune system responses, potentially increasing susceptibility to allergic conditions [10,20]. Tailored clinical interventions - such as routine dietary counseling and co-allergen testing during primary care visits - could be particularly beneficial for this subgroup.

Additionally, the highest allergy rates in the 40-59 age group suggest that food allergies in this population may persist into adulthood and develop or worsen with age, possibly due to cumulative exposure to allergens or age-related immune system changes. This pattern mirrors findings in other populations, where cumulative exposure to allergens and age-related changes in immune function contribute to developing or exacerbating allergic diseases in middle-aged adults [2,21]. Such observations highlight the importance of monitoring allergic conditions throughout life, especially for individuals in this age group who may experience delayed-onset food allergies or worsened symptoms over time.

Strengths and limitations of the study

The study's strengths lie in its comprehensive assessment of cross-reactivity, which allows for a more nuanced understanding of co-sensitization patterns that can complicate allergy management. To the best of the author's knowledge, this is the first study in Saudi Arabia to investigate the cross-reactivity prevalence and pattern among food-allergic adults. The cross-reactivity matrix generated from this study's data is useful for clinicians and dietitians to anticipate co-sensitization in patients with certain allergies, potentially enabling earlier interventions. Additionally, the demographic analysis adds depth to our understanding of food allergy trends in adult populations, revealing higher prevalence rates among females and a tendency for food allergies to be most pronounced in middle-aged adults.

However, the study has limitations that warrant consideration. First, the reliance on self-reported data may introduce recall bias; to minimize this, a structured questionnaire with a predefined checklist of common allergens was used to help guide participants' responses. Nonetheless, the possibility of underreporting or overreporting remains, particularly in a cultural context where awareness and diagnosis of food allergies may vary. Second, the use of a self-selected sample may introduce selection bias, as individuals with more severe or recognized symptoms may have been more likely to participate. Additionally, the cross-sectional design precludes conclusions about causality or the progression of sensitization over time.

Future studies could build on this work by employing longitudinal or interventional study designs and integrating objective diagnostic tools such as skin prick testing or serum-specific IgE assays to enhance accuracy and validity. Potential underreporting may also stem from limited access to specialized allergy care or a lack of awareness in certain populations, which should be considered in future research.

## Conclusions

In conclusion, the findings of this study underscore the importance of a comprehensive approach to food allergy management that considers both individual allergens and atypical cross-reactivity patterns, some of which deviate from classical IgE-mediated classifications. This approach is particularly relevant for individuals in the Makkah region who may be at elevated risk due to high cross-reactivity tendencies, as demonstrated by the cross-reactivity matrix developed in this study. This matrix can be a useful reference for clinicians and patients when anticipating co-sensitization. Moreover, these findings highlight the need for targeted clinician education on cross-reactivity mechanisms to enhance patient counseling and individualized dietary planning. Public health strategies should also consider region-specific guidelines and awareness campaigns that reflect local dietary habits and cultural factors. Future research should focus on longitudinal studies incorporating objective diagnostic tools (e.g., IgE testing and oral food challenges) to validate self-reported allergies and track sensitization patterns over time. Interventional studies assessing the impact of educational or policy interventions on allergy outcomes would also be valuable. These insights have the potential to inform not only individual patient care but also regional public health policy, improving the diagnosis, education, and quality of life for individuals with food allergies in Saudi Arabia.
